# Treatment of Chancre

**Published:** 1843-11

**Authors:** 


*Treatment of Chancre.*—There are few surgeons better acquainted with the nature and treatment of syphilis than Mr. Langston Parker, of Birmingham, and we may therefore safely recommend his mode of treating a chancre. Instead of using the nitrate of silver either solid or in solution, so as to covert the specific ulcer into a common one, (which may be done when used very early), he prefers using a much more powerful escharotic, so as at once to disorganize the tissue to a depth coequal with that of the chancre. This the nitrate of silver does not always accomplish; it often, on the other hand, converts the sore into a very irritable one, without destroying its specific character. By using a more powerful escharotic, this is more safely and certainly done; and for this purpose, highly concentrated nitric acid, the acid nitrate of mercury, the acid nitrate of silver, or the potassa cum calce of the London Pharmacopœia may be used. The second of these remedies is made by dissolving a drachm of the subnitrate of mercury in an ounce of nitric acid; the third by dissolving the same quantity of the nitrate of silver in the like quantity of acid; but Mr. Parker prefers the potassa cum calce, which is to be made into a paste of moderate consistency with spirits of wine, at the time it is wanted for use, and the sore and its edges covered with it; it may remain on from half to a minute, or even longer according to its effects. After this the caustic must be all removed by means of a fine bone spatula, and the eschar left may be covered with a poultice or any other simple application. All the parts touched by this application are at once destroyed, and on the separation of the eschar we find a clean granulating sore left. We must refer to Mr. Parker's paper for his remarks on the constitutional treatment of syphilis, but we will here shortly advert to his method of treating a bubo which we think equally judicious as his mode of destroying a chancre. If a bubo is forming, insist upon a strict regimen and absolute rest, if possible; smear the part over with mercurial ointment, over this a cold linseed poultice and a piece of oiled silk to keep it moist, confining all by a bandage. The best way, however, is to paint over the enlarged gland, night and morning, with a strong solution of iodine and hydriodate of potass; one scruple of iodine, two scruples of hydriodate of potass, and one of water. Pressure and a slight mercurial course will hasten its dispersion. If suppuration takes place, how is the abscess to be opened—not by a free incision as is commonly done, but by several very small punctures with a lancet over the thinnest part; through these the matter oozes till the cavity is empty, and a weak solution of the sulphate of zinc is then to be injected, of the strength of two or three grains to the half-pint of water. When the abscess is quite empty place a large compress over it. A weak solution of iodine may occasionally be substituted for the zinc in the following proportions: Iodine four grains, hydriodate of potass eight grains, water eight ounces. Mr. Parker thinks that the same method may be extended to the treatment of chronic abscesses.

Dr. Strohl differs materially from Mr. Parker in his treatment of chancre, and although we give his peculiar way of treating these sores, we must say that in the specific cases of syphilis we should not depend upon it. He assures us that his local treatment of chancre is one of the most successful yet practised. It consists in cauterizing the sore if it is not much inflamed nor extensive. If it is so, he employs sulphate of copper instead, and dresses the part five or six times a-day with charpie soaked in a solution of about a grain and a half of sulphate of copper to an ounce of water. The sores will generally heal in about twelve days. At other times he uses an ointment composed of two grains of cyanuret of mercury to an ounce of axunge. This ointment is spread upon a piece of linen corresponding to the size of the sore. When this is too painful, it must be taken off for a time and replaced in a weaker form. When it has been on from four to ten hours, it is dressed with mercurial ointment, or opium cerate.—*Braithwaite's Retrospect of Prac. Med. and Surg.*

